# 5-hydroxytryptamine receptor (5-HT_1D_R) promotes colorectal cancer metastasis by regulating Axin1/β-catenin/MMP-7 signaling pathway

**DOI:** 10.18632/oncotarget.4543

**Published:** 2015-07-17

**Authors:** Hua Sui, Hanchen Xu, Qing Ji, Xuan Liu, Lihong Zhou, Haiyan Song, Xiqiu Zhou, Yangxian Xu, Zhesheng Chen, Jianfeng Cai, Guang Ji, Qi Li

**Affiliations:** ^1^ Department of Medical Oncology, Shuguang Hospital, Shanghai University of Traditional Chinese Medicine, Shanghai 201203, China; ^2^ Institute of Digestive Diseases, Longhua Hospital, Shanghai University of Chinese medicine, Shanghai 200032, China; ^3^ Department of Pharmaceutical Sciences, College of Pharmacy and Health Sciences, St. John's University, Queens, NY 11439, USA; ^4^ Department of Chemistry, University of South Florida, Tampa, FL 33620, USA

**Keywords:** colorectal cancer, 5-hydroxytryptamine (5-HT) receptor, Wnt signaling pathway, metastasis, intestinal epithelium cells

## Abstract

Overexpression of 5-hydroxytryptamine (5-HT) in human cancer contributes to tumor metastasis, but the role of 5-HT receptor family in cancer has not been thoroughly explored. Here, we report overexpression of 5-HT_1D_ receptor (5-HT_1D_R) was associated with Wnt signaling pathway and advanced tumor stage. The underlying mechanism of 5-HT_1D_R-promoted tumor invasion was through its activation on the Axin1/β-catenin/MMP-7 pathway. In an orthotopic colorectal cancer mouse model, we demonstrated that a 5-HT_1D_R antagonist (GR127935) effectively inhibited tumor metastasis through targeting Axin1. Furthermore, in intestinal epithelium cells, we observed that 5-HT_1D_R played an important role in cell invasion via Axin1/β-catenin/MMP-7 pathway. Together, our findings reveal an essential role of the physiologic level of 5-HT_1D_R in pulmonary metastasis of colorectal cancer.

## INTRODUCTION

Colorectal cancer (CRC) is one of the leading causes of cancer-related human morbidity and mortality worldwide [1]. Despite recent advances in the diagnosis and therapy of CRC, the general survival rate of CRC patients has not improved [2]. Metastasis disposes patients to poor prognosis and is the main cause of mortality [3]. The molecular mechanisms underlying CRC metastasis are not quite clear to date.

Serotonin (5-hydroxytryptamine, 5-HT) is a neurotransmitter that mediates a wide variety of physiological events including central and peripheral action through the binding of multiple receptor subtypes [4]. Although the mitogenic events of serotonin receptors, especially 5-HT_1A_ receptor, 5-HT_1B_ receptor, 5-HT_1D_ receptor and 5-HT_2A_ receptor, in breast cancer, prostate cancer and bladder cancer have been investigated [5–7], there is a lack of evidence regarding whether these receptors play a significant role in CRC.

It has been found that the activation of Wnt signaling in colorectal cancer correlates with more invasive tumor growth, a higher susceptibility of disease recurrence after surgery, and a lower survival rate [8]. However, the underlying molecular mechanism of Wnt signaling is not clear. Recently, a report has shown that 5-HT_1B_ receptors on osteoblasts inhibit cell proliferation by activating PKA and CREB in Wnt signaling [9]. Although there is a great contribution of 5-HT_1_ group receptors in signaling pathway including Wnt signaling [10], there is a lack of evidence of Wnt signaling on the impact of 5-HT receptors in colorectal cancers.

In this study, we first clarified the molecular mechanism by which 5-HT_1D_ receptor (5-HT_1D_R) inhibits Wnt signaling, then we disclosed the direct effect of 5-HT_1D_R on Axin1. We demonstrated that inhibition of 5-HT_1D_R has potent anti-metastatic effect via Wnt signaling pathway. Our results provided the Wnt targeted mechanism to better understand CRC metastasis.

## RESULTS

### 5-HT_1D_R is overexpressed in CRC tumors

**Figure 1:**
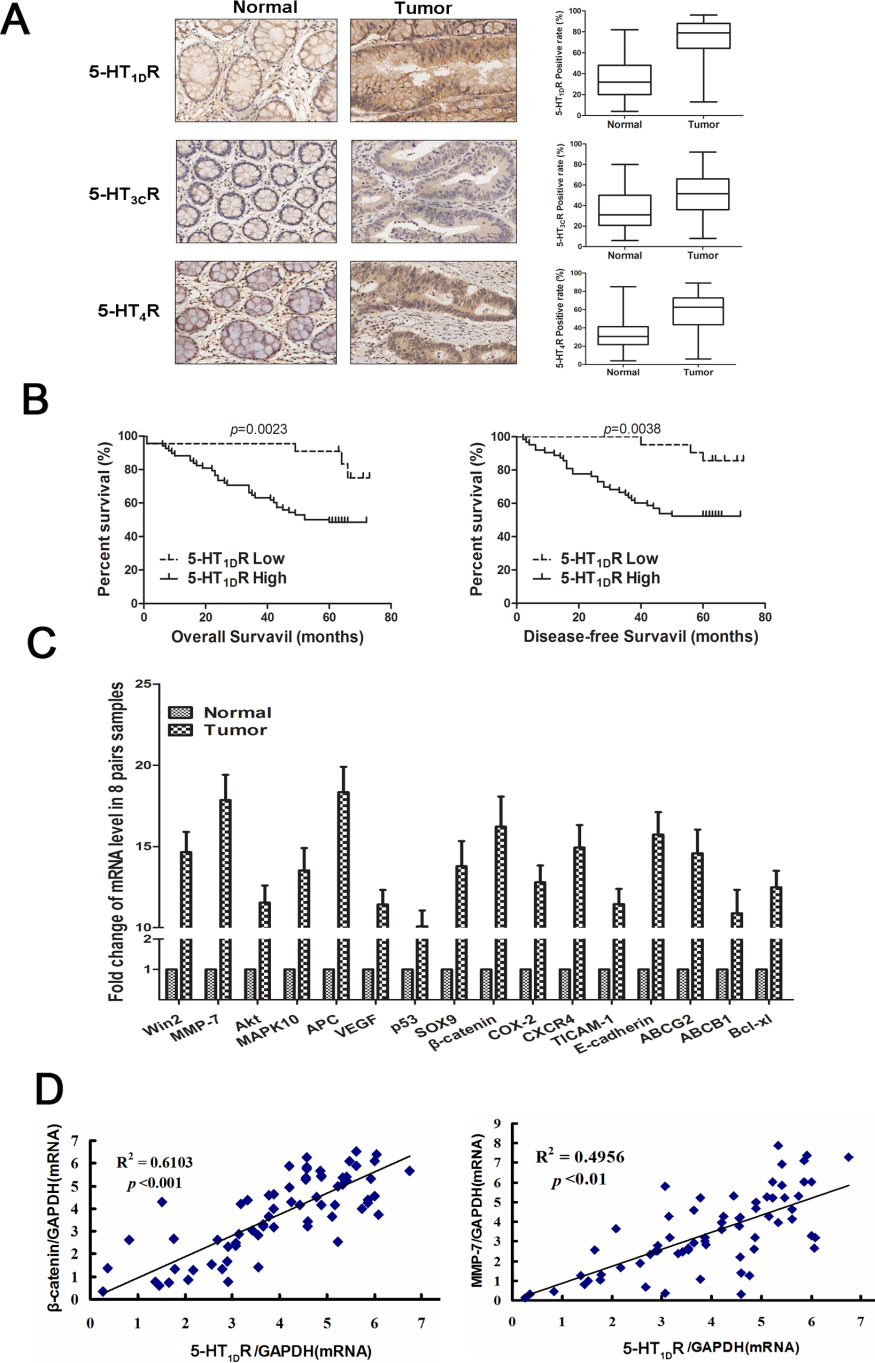
The expression of 5-HT_1D_R positively correlated with β-catenin and MMP-7 expression in human colorectal cancer tissues **A.** Left: The expression of 5-HT_1D_R, 5-HT_3C_R and 5-HT_4_R protein was evaluated by immunohistochemistry. Right: 5-HT_1D_R, 5-HT_3C_R and 5-HT_4_R protein expression quantification exemplified in 90 primary CRC tissue samples and matched with normal tissue. **B.** OS and DFS curves for all studied patients with high or low 5-HT_1D_R expression (*n* = 90). **C.** Full cDNA microarray analysis is performed to detect the different expression of cancer genes in 8 pairs of patient-matched normal tissues with a positive 5-HT_1D_R expression. **D.** Left: Correlation analysis between the relative mRNAs of 5-HT_1D_R and β-catenin in 5-HT_1D_R positive human colorectal tumors (*n* = 68). Right: Correlation analysis between the relative mRNAs of 5-HT_1D_R and MMP-7in 5-HT_1D_R positive human colorectal tumors (*n* = 68). (Spearman correlation test)

To study the expression pattern of HTR in colorectal cancer, the protein level of 5-HT_1_R, 5-HT_2_R, 5-HT_3_R and 5-HT_7_R were quantified by immunohistochemical staining in 90 pairs of colorectal cancers and matched adjacent normal colon tissue samples. Upregulation of 5-HT_1D_R, 5-HT_3C_R and 5-HT_4_R protein level was observed in colorectal cancer samples compared with the paired normal tissues (Figure 1A). Notably, the expression of 5-HT_1D_R (68 out of 90, 76%) was observed being the highest among all 5-HT receptors. Then we divided the patients into two groups: high level of 5-HT_1D_R (68) and low level of 5-HT_1D_R (22). Statistical analysis indicated that patients with high level of 5-HT_1D_R had significantly worse overall survival (OS) and disease-free survival (DFS) compared with patients with low level of 5-HT_1D_R (Figure 1B). This was not associated with sex, age, tumor grade, carcino-embryonic antigen (CFA) and fetoprotein (AFP) level (Supplementary Table S1). The 5-year OS was 48.8% for high level of 5-HT_1D_R and 64.7% for low level of 5-HT_1D_R patients (*p* = 0.0023). The 5-year PFS was 39.9% for high level of 5-HT_1D_R and 56.2% for low level of 5-HT_1D_R patients (*p* = 0.0038).

To further characterize the role of 5-HT_1D_R overexpression in colorectal cancer, we performed a full cDNA microarray to screen for different expression of cancer genes in 7 pairs of patient-matched normal tissues (data not shown). As viewed in Figure 1C and Supplementary Figure S1, a total of 16 genes were differentially upregulated (by more than 10 times), and involved seven signal pathways. Interestingly, components of the Wnt signaling pathway, including MMP-7, β-catenin, and APC, were significantly upregulated in high 5-HT_1D_ R tumor tissue, implying a possible link between 5-HT_1D_ R and Wnt signaling pathway in CRC patients.

Importantly, in 68 CRC patients with high level of 5-HT_1D_R, a positive and significant association between 5-HT_1D_R and c-myc or MMP-7 gene was observed (Figure 1D), whereas no such correlation was seen in the other 14 genes. MMP-7 is a known downstream target of Wnt signaling pathway and protein MMP-7 can be upregulated when the Wnt signaling pathway are activated. Collectively, our results suggest a possible link between 5-HT_1D_R upregulation and Wnt/MMP-7 signaling pathway in CRC progression.

### 5-HT_1D_R competitively bound to Axin1 and released Axin1 from the destruction complex

**Figure 2:**
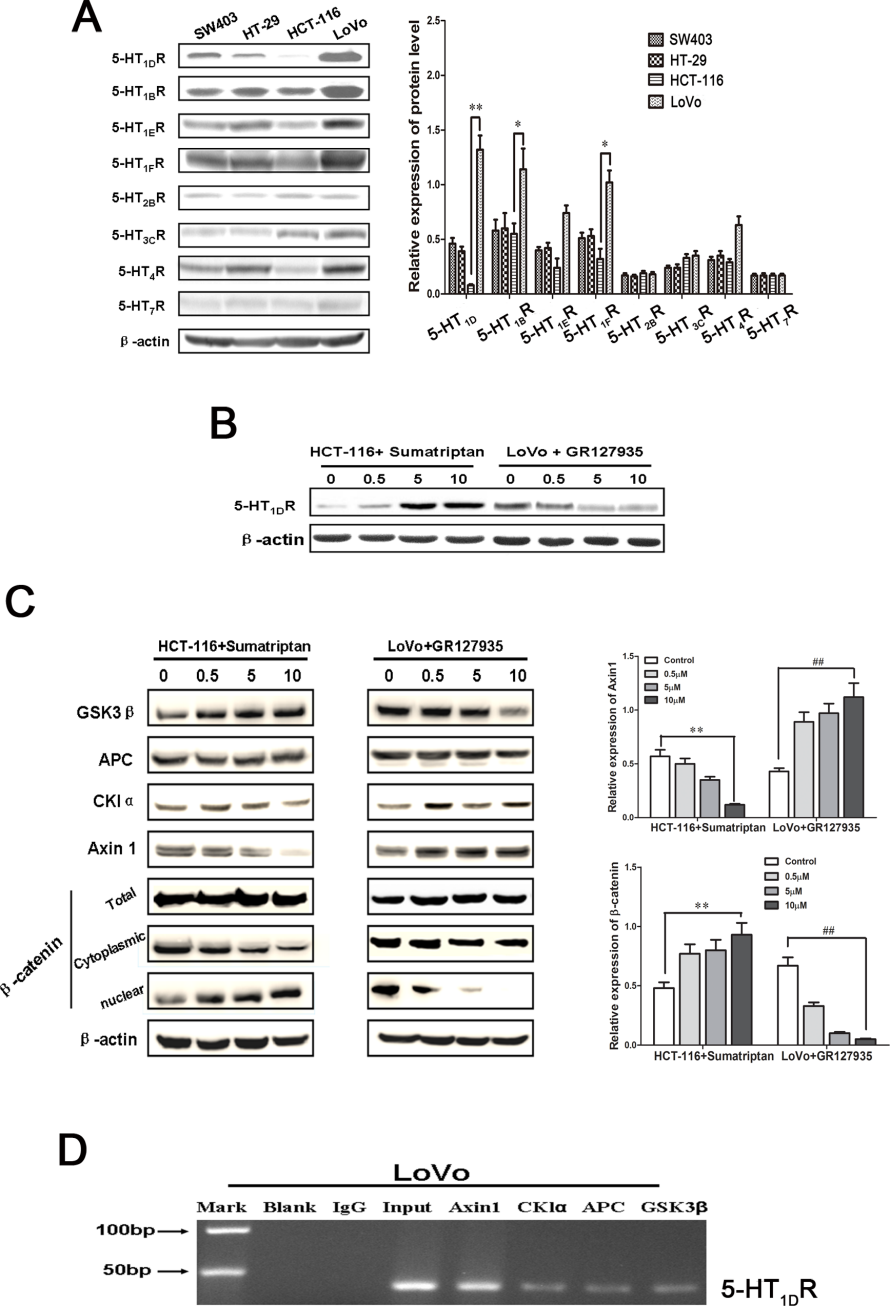
5-HT_1D_R competitively bound to Axin1 and regulated pivotal signaling transduction pathways **A.** Left: The expression of 5-HTR family members in 4 human CRC cells (LoVo, HCT-116, HT-29 and SW403). Right: Quantification of the relative protein expression. Values are mean ± SEM. (significant differences are indicated by *P < 0.05, **P < 0.01). **B.** Western blot analysis of 5-HT_1D_R expression levels in HCT-116 and LoVo cells treated with 5-HT_1D_R agonist (sumatriptan) and antagonist (GR127935) for 48 h. **C.** Left: Western blot analysis of APC, Axin1, GSK3β, CKIα and β-catenin expression after HCT-116 and LoVo cells were treated with sumatriptan and GR127935 for 48 h. Right: Quantification of the relative Axin1 and β-catenin expression. Values are mean ± SEM. (significant differences are indicated by **P < 0.01, ^##^*P* < 0.01). **D.** ChIP analysis between 5-HT_1D_R protein and APC, Axin1, GSK3β, CKIα gene. As a control, mouse monoclonal IgG was used. The blank group was the PCR results with no cDNA, and the input group was the cDNA from cell lysates without RIP procedure. The mean and standard errors from triplicate experiments are indicated.

Western blotting analysis revealed that the family members of 5-HTR protein were differentially expressed in 4 human CRC cells (LoVo, HCT-116, HT-29 and SW403) (Figure 2A). As we know, LoVo cells are a well differentiated cells. Interestingly, 5-HT_1D_R, 5-HT_1B_R and 5-HT_1F_R were more overexpressed in LoVo cells than that in HCT-116 cells, which are poor differentiated cells. Since previous evidence indicated that targeting 5-HT_1D_ receptor subsequently targets Wnt pathway, we investigated which factor is involved in 5-HT_1D_R regulating Wnt pathway. First, using sumatriptan to increase the level of 5-HT_1D_R, we found the expression of 5-HT_1D_R in HCT-116 was upregulated, whereas 5-HT_1D_R protein was downregulated in LoVo cells in a dose-dependent manner after treatment with GR127935 (Figure 2B). Second, to verify the effect of 5-HT_1D_R on canonical Wnt/β-catenin signaling pathway, other components of the “destruction complex” including APC, Axin1, GSK3β and CKIα were tested. As shown in Figure 2C, when sumatriptan was applied, the Axin1 level was markedly decreased in HCT-116 cells, but increased in LoVo cells (Figure 2C and right-upper). Consistent with our findings in Axin1 expression, β-catenin protein showed a decreased expression in the cytoplasm of HCT-116 cells, but an increased expression in the HCT-116 nucleus in a dose-dependent manner (Figure 2C). These findings suggest that 5-HT_1D_R is a gene involved in the response to nuclear accumulation of β-catenin in activation of the Wnt/β-catenin pathway. As protein interaction is the first step for epigenetic regulation, we then checked whether 5-HT_1D_R is associated with Axin1 by ChIP analysis. Among the three members APC, Axin1, GSK3β and CKIα of the destruction complex family, only Axin1 co-precipitated with 5-HT_1D_R in 5-HT_1D_R overexpression LoVo colorectal cancer cells (Figure 2D).

### 5-HT_1D_R regulated Axin1/β-catenin/LEF1/TCF4/MMP-7 signaling

**Figure 3:**
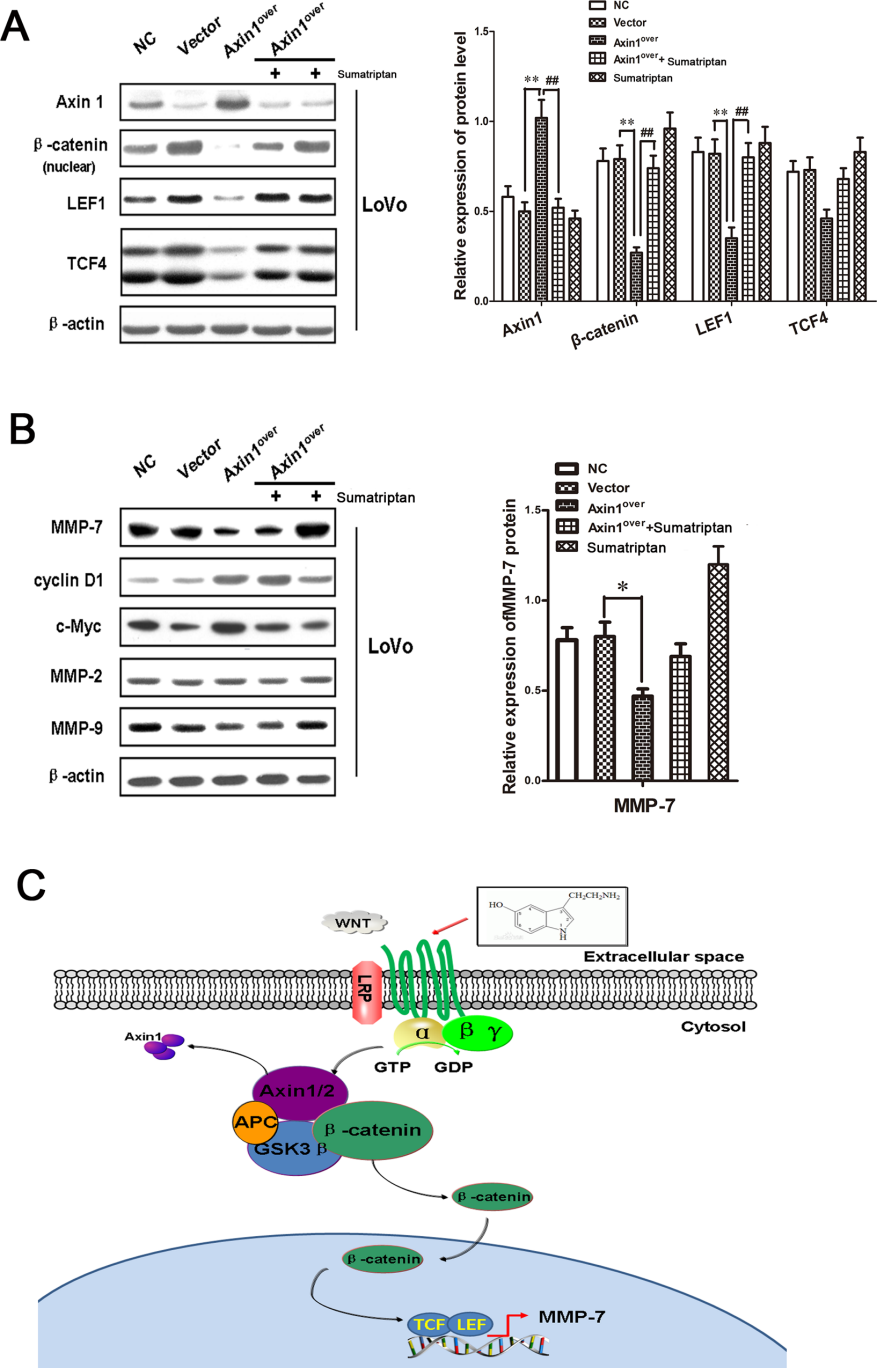
5-HT_1D_R regulated Axin1/β-catenin/LEF1/TCF4/MMP-7 signaling pathway **A & B.** Western blots showing expressions of Axin1, β-catenin, LEF, TCF4, MMP-7, cyclin D1, c-Myc, MMP-2 and MMP-9 in response to transfection with lentivirus-control (Vector), Axin1 lentivirus (Axin1^over^), 5-HT_1D_ agonist or co-transfection with sumatriptan and Axin1 lentivirus (Axin1^over^) for 48 h as described in Materials. Quantification of the relative Axin1, β-catenin, LEF, TCF4, MMP-7 expression. Values are means ± SEM. (significant differences are indicated by *P < 0.05, **P < 0.01). **C.** Proposed working model of the noted 5-HT_1D_R lead of Axin1/β-catenin/LEF1/TCF4/MMP-7 signaling pathway mediated invasion and migration in colorectal cancer cells.

Since LEF1/TCF4 is one of the major downstream of Wnt/β-catenin pathway, we hypothesized that 5-HT_1D_R may utilize Axin1/β-catenin/LEF/TCF4 to influence its metastatic effects. We evaluated the level of LEF/TCF4 in LoVo cells with or without sumatriptan treatment after transfected with Axin1-overexpressing vector. As expected, the level of LEF/TCF4 was significantly upregulated in Axin1-overexpressing LoVo cells compared to that of LoVo cells transfected with empty vector (Figure 3A). Importantly, the combination of sumatriptan and Axin1-overexpressing vector offset the inhibitory effect of Axin1, suggesting that 5-HT_1D_R regulates the expression of Wnt/β-catenin/LEF/TCF4 specifically through Axin1 separated from the destruction complex. To further clarify the molecular mechanisms of the 5-HT_1D_R activation of the Axin1/β-catenin/LEF/TCF4 pathway, we compared the expression profiles of Wnt-related molecules in LoVo cells including MMP-7, cyclin D1, c-Myc, MMP-2 and MMP-9. Western blot result showed that only expression of MMP-7 changed with a similar trend as the level of LEF1/TCF4 (Figure 3B). The findings from this study revealed an inherent role of 5-HT_1D_R which binds Axin1 and dissociates β-catenin from the complex, moving β-catenin into the nucleus to activate the β-catenin/LEF1/TCF4/MMP-7 pathway in colorectal cancer metastasis (Figure 3C).

### 5-HT_1D_R modulates colorectal cancer invasion/migration *in vitro*

**Figure 4:**
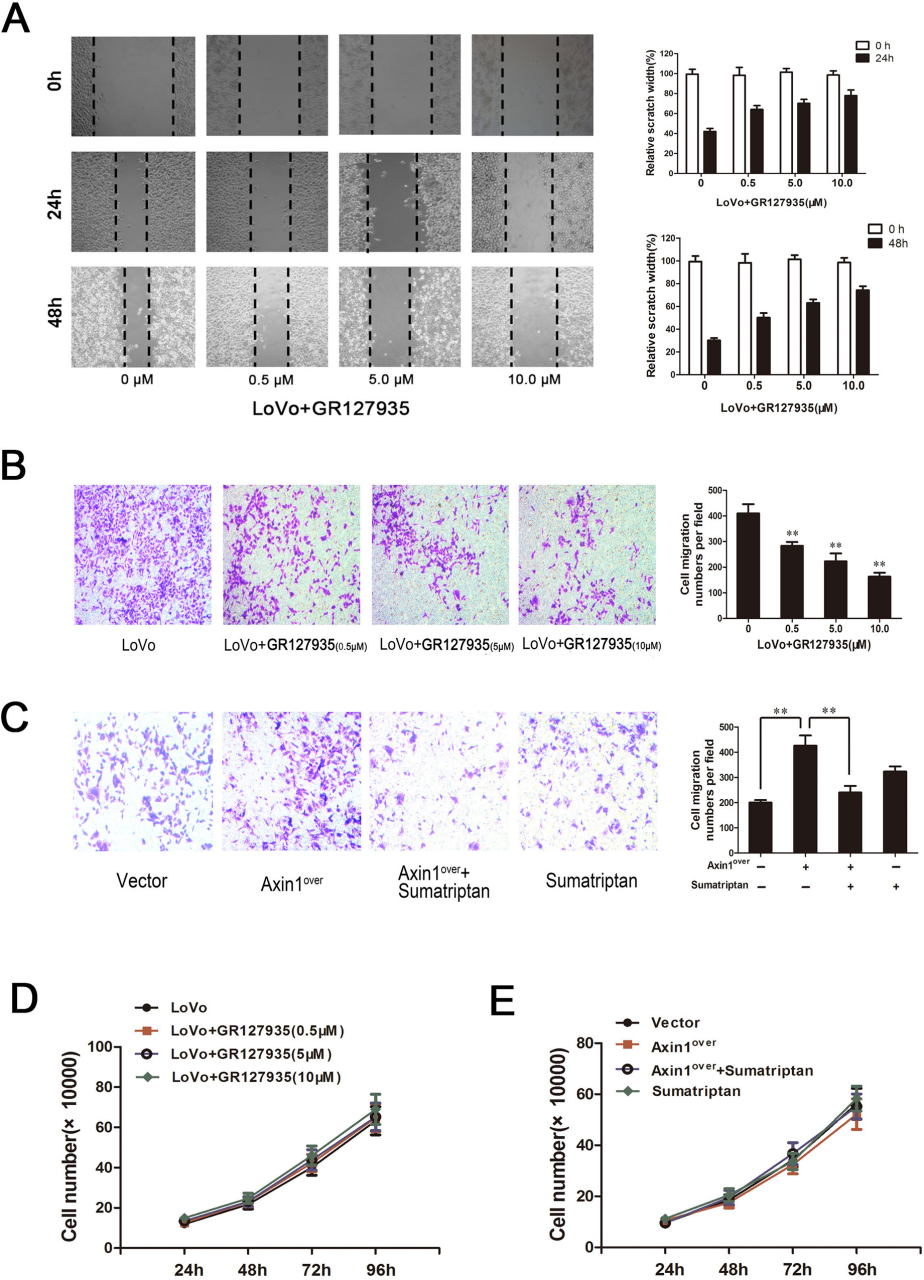
5-HT_1D_R modulated invasion and migration in colorectal cancer cell **A & D.** Wound-healing assay. Images were taken 0, 24 and 48 hours after wound formation. Data are presented as mean ± SD of triplicate experiments.*P < 0.05, **P < 0.01 ^##^*P* < 0.01 in Fig. 4D. **B & C.** Cell invasion assay result using Matrigel-coated Transwell. (left, representative pictures of invasion chambers; right, average counts from five random microscopic fields). Data are presented as mean ± SD of triplicate experiments. *P < 0.05 *vs*. Vector group in Fig. 4A; *P < 0.05, **P < 0.01 in Fig. 4B. **D.**The cell proliferation of GR127935-treated LoVo cells in different concentration at 24 h, 48 h, 72 h, 96 h as described in Materials. **E.** Cell proliferation was assayed at 24, 48, 72 and 96 hours after LoVo cells were transfected with Axin1 lentivirus (Axin1^over^) or treated with sumatriptan or treated with both together.

To determine whether the 5-HT_1D_R may play a role in tumor invasion, GR127935 was applied to LoVo cells and analyzed using a scratch-wound motility assay. The data in Figure 4A showed a time and dose-dependent downregulation of cell migration in response to GR127935 treatment, as shown by as much as 40% delay in wound closure at 48 hours post-treatment with the highest dose of GR127935 (Figure 4A right). Similar results were seen in Matrigel-coated Transwell assays, in which LoVo cells were treated with GR127935 in a dose-dependent manner (Figure 4B). In addition to the offset effect of sumatriptan to Axin1, we also elucidate the role of sumatriptan in Axin1-mediated cell migration. Transfection of Axin1-overexpressed vector and a specific sumatriptan dose were used to inhibit the activity of Axin1 mediated signaling pathway in LoVo cells. As expected, compared with vector cells, Axin1-overexpressing cells showed more migration activity; however the activity was decreased after adding sumatriptan (Figure 4C). Finally, we evaluated cell viability after GR127935 treatment with the trypan blue exclusion assay. As shown in Figure 4D and 4E, the proliferation and migration of LoVo cells remained unchanged, implying that 5-HT_1D_R plays a key role in the regulatory effect of Axin1-mediated cell migration without the proliferation change.

### 5-HT_1D_R modulates colorectal cancer invasion/migration *in vivo*

**Figure 5:**
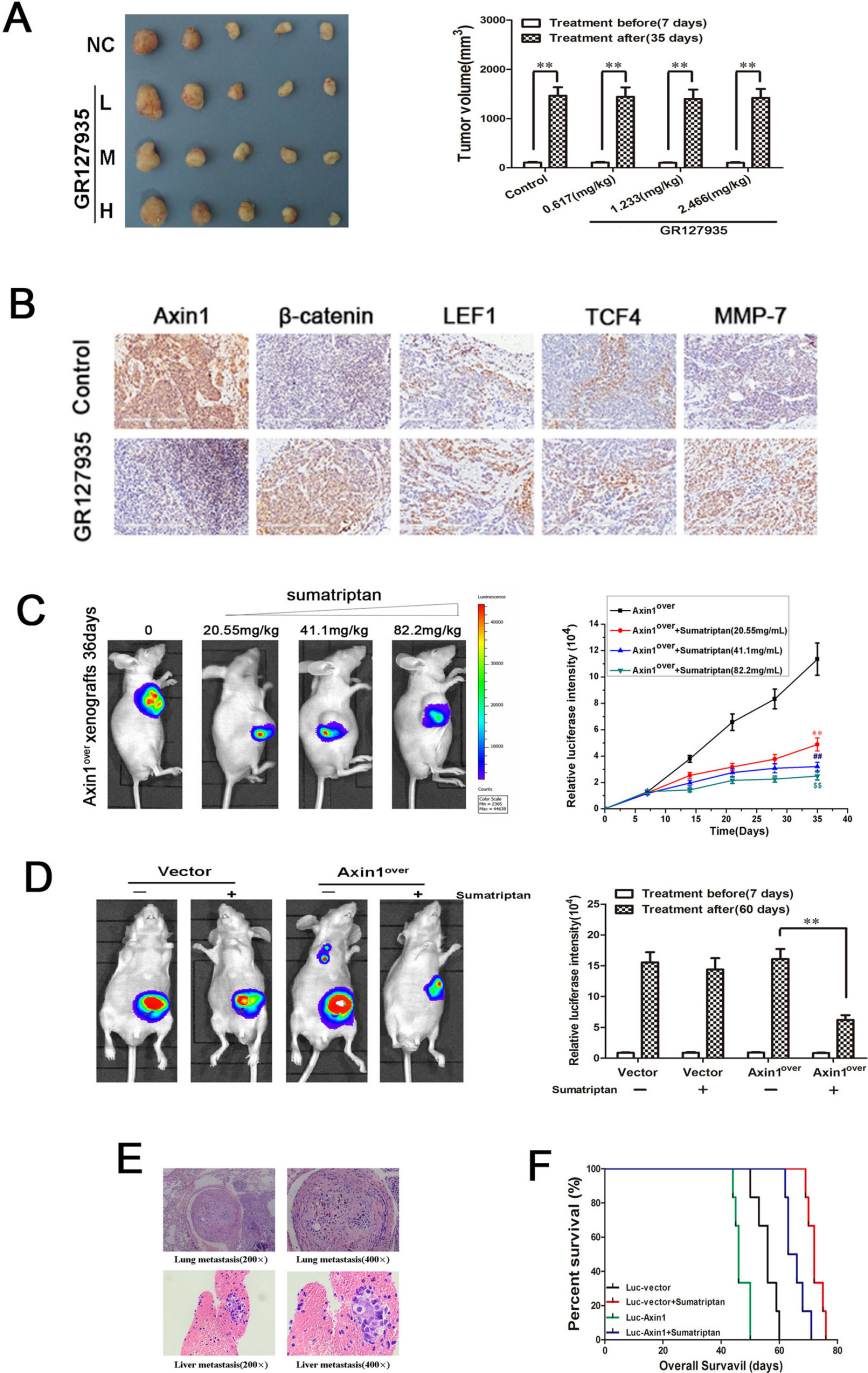
5-HT_1D_R modulated colorectal cancer invasion/migration *in vivo* **A.** Excised tumors on day 28 of treatment with GR127935 (35 days after inoculation). Tumors were weighed (right) and photographed (left). Data are means ± SEM. Significant differences are indicated by **P < 0.01 (representative one in each group has been shown). **B.** The subcutaneous transplanted tumor tissue in Fig. 5A were subjected to immunohistochemical analysis using Axin1, β-catenin, LEF1, TCF4 and MMP-7 antibody. Images were taken at 200x magnification. Brown staining indicated positive cells. **C.** Left: Luciferase imaging of the mice with Axin1 overexpression and sumatriptan xenografts on day 36 after tumor cell implantation. Right: the quantification of luciferase intensities in tumors of the four groups. Data are presented as mean ± SD of triplicate experiments. **P < 0.01 sumatriptan (0.617 mg/kg) *vs*. Control. ^##^*P* < 0.01 sumatriptan (1.233 mg/kg) *vs*. Control. ^$$^*P* < 0.01 sumatriptan (2.466 mg/kg) *vs*. Control. **D.** Luciferase imaging revealed primary tumor growth and its distant metastasis. Right: the quantification of luciferase intensities of the mice treated with sumatriptan for 60 days. Data are means ± SD. significant differences are indicated by **P < 0.01. **E.** H&E analyses of lung and liver sections in Axin1 overexpressing orthotopic implanted animals by day 60, which was the only group with distant metastasis. **F.** overall survival of orthotopic model is shown in Fig. 5D.

To further test our hypothesis of 5-HT_1D_R's role in migration and invasion *in vivo*, we first established a subcutaneous xenograft tumor model of colorectal cancer cell line. Compared with normal saline group, the tumor growths were not apparent after treatment with GR127935 (Figure 5A). The toxicity of GR127935 was evaluated by observing the body weight, and no significant weight loss was observed (Supplementary Figure S2). However, immunohistochemistry analyses of Axin1 displayed a downregulation in the tumor of mice treated with GR127935 compared with that of the control group. Similar to the result *in vitro*, the levels of β-catenin, LEF1, TCF4 and MMP-7 increased after treatment with GR127935 compared with those of the control group (Figure 5B). Next, we generated a xenograft tumor model using fluorescence-labeled Axin1-overexpressed colorectal cancer cell. When we treated these tumor-bearing mice with sumatriptan, the fluorescence activity of Axin1 decreased in a dose-dependent manner (Figure 5C). We also used fluorescently-labeled Axin1-overexpressed cells in a colonic orthotopic xenograft. After sumatriptan treatment for 28 days, the fluorescent activity of Axin1 was significantly attenuated compared with that of the control group (Figure 5D). Furthermore, we examined the invasion and migration by hematoxylin and eosin (H&E) staining in main organ tissue slices. We found more colonic metastases in liver and lung tissue in Axin1-overexpressed group, which were decreased in Axin1-overespressed group treated with sumatriptan (Figure 5E). Corresponding survival curves showed a significantly increase in median survival of sumatriptan-treated group. We also saw that the luc-vector group had significantly prolonged overall survival than the Axin1-overexpressed group with or without sumatriptan (Figure 5F).

### 5-HT_1D_R modulates colorectal cancer invasion/migration in IECs

**Figure 6:**
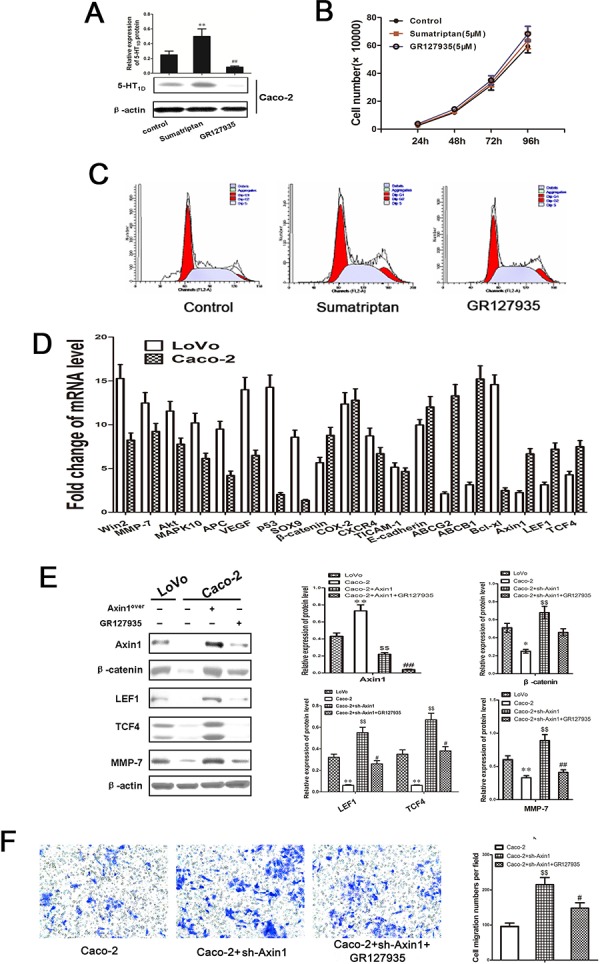
5-HT_1D_R modulated colorectal cancer invasion/migration in IECs **A.** Relative expression levels of 5-HT_1D_R were detected by Western blot in Caco-2 cells treated with sumatriptan and GR127935. **B.** The cell proliferation of sumatriptan and GR127935 treated Caco-2 cells in different concentrations at 24 h, 48 h, 72 h, and 96 h. **C.** Flow cytometry analysis was used to distinguish cells in different phases of the Caco-2 cell cycle as described in Methods. **D.** The mRNA levels of 16 genes were quantified by qRT-PCR in LoVo and Caco-2 cell**. E.** Western blots showing expression levels of Axin1, β-catenin, LEF, TCF4, and MMP-7 in LoVo and Caco-2 cell infected by Axin1 lentivirus (Axin1^over^), co-treated with GR127935 and Axin1 lentivirus (Axin1^over^) for 48 h as described in Materials. Data are presented as mean ± SD of triplicate experiments. **P < 0.01 Caco-2 *vs*. LoVo; ^$$^*P* < 0.01 Caco-2+Axin1^over^ *vs*. Caco-2; ^##^*P* < 0.01 Caco-2+Axin1^over^+GR127935 *vs*. Caco-2+Axin1^over^. **F.** Caco-2 cell invasion assay result using Matrigel-coated Transwell. The bold horizontal bar represents mean expression levels; Data are presented as mean ± SD of triplicate experiments. ^$$^*P* < 0.01 Caco-2+Axin1^over^ *vs*. Caco-2; ^#^*P* < 0.05 Caco-2+Axin1^over^+GR127935 *vs*. Caco-2+Axin1^over^

Because increased motility and invasion are among the principal effects of upregulated Axin1/β-catenin signaling, those features were tested in 5-HT_1D_R overexpressing colorectal cancer cells. To test whether 5-HT_1D_R also regulate motility and invasion via Axin1/β-catenin signaling in intestinal epithelium cells, we use Caco-2 cell as intestinal epithelium cells, and measured the level of 5-HT_1D_R with or without sumatriptan and GR127935. Western blot results showed a low level of 5-HT_1D_R in Caco-2 cell, and sumatriptan or GR127935 could increase or decrease the level of 5-HT_1D_R respectively (Figure 6A). Next, we assessed its toxicity in Caco-2 cells. As shown in Figure 6B, sumatriptan or GR127935 treatments do not significantly inhibit cell growth or slow the rate of Caco-2 cell cycle (Figure 6C). To investigate the potential mechanism of Caco-2 cells and LoVo cells, we used PCR to examine the expression of mRNA in 19 genes including their difference in the tumor sample previously found and Axin1, LEF1, TCF4. As shown in Figure 6D, Axin1, β-catenin, LEF, TCF4 and MMP-7 were downregulated in Caco-2 cells. Furthermore, we also tested the effect of sumatriptan in the Axin1/β-catenin-related signal pathway, which has been verified in LoVo cells. Figure 6E and 6F showed that 5-HT_1D_R may utilize Axin1/β-catenin/LEF/TCF4 to influence its metastatic effects.

## DISCUSSION

Cancer invasion and metastasis are complex processes involving increase in cell motility, adhesion to underlying stroma, digestion of extracellular matrix, vascular invasion, evasion of immune attack, and growth in foreign environments [17–19]. One way to identify the genes involved in these processes is to compare the gene expression profile between normal and tumor tissue. In the present study, 5-HT_1D_R was shown to be a significantly overexpressed in the human colorectal cancer sample (76%), which was assumed to drive poor outcome in large patient cohorts. Moreover, we found 16 genes upregulated in 7 pairs of 5-HT_1D_R overexpressing tumor compared to their normal tissues by genome-wide microarray analysis. Similar to studies of hepatocellular carcinoma [20], approximately 30.8% of all patients showed high expression of CTHRC1 and this was associated with a lower 10-year overall and disease-free survival rates. Collectively, our results suggest that 5-HT_1D_R is a promising target in clinical therapy of patients with advanced CRC. Unfortunately, the molecular mechanisms underlying 5-HT_1D_R in CRC metastasis are still unclear.

Wnt pathway is known as a “destruction complex” in which Axin and adenomatous polypopsis coli (APC) form a scaffold facilitating β-catenin phosphorylated by CK1a and GSK3β at serine and threonine residues in its amino-terminal region [21, 22]. In a normal steady state, the cells are not exposed to Wnt signaling [23, 24]. However, activation of Wnt/β-catenin signal transduction pathway is thought to lead to a higher susceptibility of disease recurrence after surgery and a lower survival rate [25, 26]. Its mechanism is illustrated as follows: Axin dissociates from the APC/Axin/GSK/β-catenin-complex, then β-catenin translocates to the nucleus where it binds to T-cell factors (Tcf) and activates the transcription of specific target genes, including c-Myc, cyclin D1 and MMP7 [27, 28].

Because of the demonstrated relationship between 5-HT_1D_R, Wnt/β-catenin and MMP-7 from clinical data analysis, we hypothesized that 5-HT_1D_R may be involved in Wnt/β-catenin/MMP-7 mediated CRC metastasis. We increased 5-HT_1D_R expression in HCT-116 cell using an agonist, and revealed a concomitant reduction in Axin1 protein level and a concomitant increase of β-catenin protein level in the nucleus. On the contrary, GR127935 had the opposite effect in LoVo cells. Previous studies have illustrated the separation of “destruction complex” leading to an increased in Axin and [β-catenin/TCF] signaling in the cellular environment [29, 30]. Although APC and GSK3β are reported to be components of the “destruction complex”, we did not find significant changes in the expression levels of APC or GSK3β after the two colorectal cancer cells treatment with sumatriptan or GR127935. Therefore, it seems that highly-differentiated cells have an intrinsic mechanism to target Axin1 and activate β-catenin/TCF signaling pathway [31], which is still maintained when the overall level of 5-HT_1D_R is elevated in cancer cells. Furthermore, the ChIP assay also confirmed that the 5-HT_1D_R protein could bind to the Axin1 gene promoter in LoVo cells, but not to APC or GSK3β. Additionally, the results also showed that 5-HT_1D_R did not affect the other gene, only changed the level of MMP-7. CTHRC1 was also reported to be an activator of the planar cell polarity pathway of Wnt signaling in mouse embryogenesis [32]. In this work, we also provide some insights into the biological effects of overexpression of 5-HT_1D_R in CRC. Our *in vitro* data further showed that GR127935 functions as a tumor suppressor via Axin1/β-catenin/MMP-7 pathway in the cell cycle, proliferation, and invasion. This is the first work that we know of to use a mouse model system of Axin1-overexpression on the primary colon tumors to identify the effect of 5-HT_1D_R in metastasis.

There are indications that some additional genetic or epigenetic alterations are required in normal cells, thereby leading to a gain-of-function phenotype. In agreement with this possibility, our results in the normal primary colon cell, as well as studies by others report, show that the activation of Axin1/β-catenin/MMP-7 pathway is acquired only after 5-HT_1D_R activation.

We reported in our study that 5-HT_1D_R activated Axin1/β-catenin/MMP-7-mediated signaling by targeting Axin1 directly induces potent invasion and migration activity both *in vitro* and *in vivo*. The correlation between 5-HT_1D_R expression and Axin1 further provided a mechanism-based rationale to target Axin1. In conclusion, we suggest that Axin1/β-catenin/MMP-7 may be a useful prognostic marker for CRC progression and in determining the sensitivity of 5-HT_1D_R as a potential target for patients with CRC.

## MATERIALS AND METHODS

### Cell culture and reagents

The human colorectal cancer LoVo, HCT-116, HT-29 and SW403 cell lines were purchased from the Shanghai Cell Collection (Shanghai, China). Cells were grown in RPMI 1640 medium supplemented with 10% (v/v) heat-inactivated fetal calf serum, 2 mM glutamine, 100 units/ml penicillin, and 100 μg/ml streptomycin (Invitrogen, Carlsbad, CA) at 37°C in a 5% CO_2_-humidified atmosphere. Monoclonal antibodies against 5-HT family, Axin1, β-catenin, APC, GSK3β, CKIα, LEF1, TCF4, cyclin D1, c-Myc, MMP-2, MMP-9, MMP-7 and β-actin were products of Cell Signaling Technology (Beverly, MA, USA). 5-HT_1D_R agonist (Sumatriptan) and 5-HT_1D_R antagonist (GR127935) were from Sigma Chemical Co. (St. Louis, MO).

### Clinical samples

From 2008 to 2014, unifocal, primary CRC biopsies surgically resected from 90 patients, who received detailed pathological assessment and regular follow-up at Shuguang Hospital Shanghai University of Traditional Chinese Medicine were collected for this study. The basic clinical characteristics of the 90 patients are presented in Supplementary Table S1. All of the donors or their guardians provided written consent and ethics permission was obtained for the use of all samples. This study was approved by the Medical Ethics and Human Clinical Trial Committee of the affiliated hospitals, Shanghai University of Traditional Chinese Medicine.

### Immunohistochemical analysis

The hydrated paraffin section were incubated in a blocking solution (10% donkey serum +5% nonfat dry milk +4% BSA +0.1% Triton X-100) for 10 min, and then incubated at 4°C overnight with anti-5-HT family antibody. After washing with PBS, the sections were incubated with diluted (1:200) biotinylated secondary antibody for 30 min. Subsequently, the sections were washed again in PBS and incubated for 30 min with the preformed avidin-horseradish peroxidase macromolecular complex. Development of peroxidase reaction was achieved by incubation in 0.01% 3, 3-diaminobenzidine tetrahydrochloride (DAB) in PBS containing 0.01% hydrogen peroxide for approximately 5 min at room temperature. Sections were then washed thoroughly in tap water, counterstained in haematoxylin, dehydrated in absolute alcohol, cleared in xylene and mounted in synthetic resin for microscopic examination.

### Transfection of lentivirus

Transfection procedures were performed according to manufacturers' instructions, with Lipofectamin 2000 as transfection reagent (Invitrogen). Axin1-GFP/luciferase lentivirus and control GFP/luciferase lentivirus were used to transfect LoVo cells. In brief, 2 × 10^5^ cells were plated in each well of a 24-well plate and incubated overnight. Lentiviral supernatants were added to cells according the concentration of lentivirus for 12 h. The GFP positive cells were sorted and the stable clones were isolated.

### Analysis of gene expression

Total RNAs were reverse-transcribed to cDNA using the Advantage RT-PCR Kit (Clontech Laboratories, Inc., MountainView, CA, USA). For quantitative real-time PCR (qRT-PCR), target gene expression was normalized to glyceraldehyde-3-phosphate dehydrogenase expression. Primers are described in online Supplementary Table S2.

### Western blot analysis

Whole cell lysates for Western blot analysis of 5-HTR family, Axin1, β-catenin, APC, GSK3β, CKIα, LEF1, TCF4, cyclin D1, c-Myc, MMP-2, MMP-9, MMP-7 and β-actin expression were prepared as previously reported [11]. Briefly, the cells were lysed on ice in immunoprecipitation assay buffer for 2 h before being homogenized using a mortar and pestle. The homogenized sample was centrifuged, and the supernatant was collected and stored at −80°C until used. Densitometric analysis was performed using the Scion Imaging software (Scion Corporation), with β-actin as internal reference.

### Immunoprecipitation

Immunoprecipitation was performed as described previously [11], except N-ethylmaleimide was added to the cell lysis buffer at a final concentration of 10 mM to preserve poly-ubiquitinated protein conjugates.

### Cell viability assay

Cells were stained with 2 mg/ml propidium iodide as described previously [12]. Events in live cell gate were counted by an FACS Calibur. Ratio of live to total events was calculated as percent viability. All experiments were done with 5 replicates per experiment and repeated at least 3 times.

### Cell cycle analysis

Cells were treated with 100 nM Dasatinib or DMSO for 24–72 h. Cell cycle was determined using the FITC BrdU Flow kit (BD Biosciences, San Diego, CA, USA) following the manufacturer's instructions [13]. Subsequently, cells were washed once with PBS and analyzed by FACS. The percentage of cells in various phases of the cell cycle, namely, G1, G2, and S, were analyzed using ModFit software.

### Cell invasion and wound healing assays

The Matrigel invasion assay was done using the BD Biocoat Matrigel Invasion Chamber (pore size: 8 mm, 24-well; BD Biosciences, USA) following the manufacturer's protocol [14]. From five randomly selected fields, the invading cells were counted under a light microscope. For wound-healing assay, cell monolayers were scratched with a clean pipette tip and cell migration was observed for up to 24 h.

### Animals and xenograft models

Male athymic nude mice (NCr-nu), 8–12 weeks old, were purchased from Sino-British SIPPR/BK lab Animal Co., Ltd (Shanghai, China, license No. SCXK 2008-0016), and maintained under specific-pathogen-free conditions. All animal protocols were approved by the Institutional Animal Use and Care Committee. All the experiments and animal care were approved by Shanghai Medical Experimental Animal Care Commission and in accordance with the Provision and General Recommendation of Chinese Experimental Animals Administration Legislation

For experiment 1, 24 mice were subcutaneously injected with 1.0 × 10^6^ LoVo cells per animal. When the tumors reach an average size of 100 mm^3^, the mice were randomized into 4 groups (*n* = 6 per group) and received intragastric administration of 5-HT_1D_R antagonist (GR127935) at the doses of 0 mg/kg, 0.617 mg/kg, 1.233 mg/kg and 2.466 mg/kg every day as previously described [15]

For experiment 2, 24 mice were subcutaneously injected with Axin1-overexpressed luciferase LoVo cells per animal. When the tumors reach an average size of 100 mm^3^, the mice were randomized into 4 groups (*n* = 12 per group) and received intragastric administration of sumatriptan at the doses of 20.55 mg/kg, 41.1 mg/kg and 82.2 mg/kg. In clinical practice, 5-HT_1D_R agonist (Sumatriptan) is usually prescribed at a maximum daily dose of 200 mg. When this human dose is converted into an animal dose (a person of 60 kg, and a conversion factor of 12.33 between human and mouse), it was equivalent to the middle dose (41.1 mg/kg) used in this study. Six mice were sacrificed in each group on the 28th day after treatment (35 day after transplantation), the other 6 mice in the same group were observed longer for survival time

For experiment 3, 24 mice underwent colon orthotopic transplantation with Axin1-overexpressed luciferase or vector luciferase LoVo cells per animal. The treatment was similar to experiment 2, except that the time of intragastric administration of sumatriptan started from 30 days after transplantation due to a prior observation that metastasis of colorectal cancer needs at least one month to take place [16]. Six mice were sacrificed in each group on the 60th day after transplantation, the other 6 mice in the same group were observed longer for survival time. The body weight of the animals and the two perpendicular diameters (A and B) were recorded every 3 days and tumor volume (V) was estimated according to the following formula [16] :V = π/6 × [(A+B)/2]^3^. The survival time for each group and overall significance was plotted on a Kaplan-Meier survival curve using GraphPad Prism

### Bioluminescence imaging

Bioluminescence imaging and data acquisition were performed using D-luciferin potassium salt and the IVIS 100 imaging system coupled to the Living Image software (Xenogen) as previously reported [2].

### H & E staining

The colon tissues containing primary tumors, liver and lungs harvested from CRC xenograft tumor group 5 and 6 mice were explanted, imaged, and immediately fixed in 10% neutral buffered formalin for 24 h. The tissues were then processed, embedded in paraffin, and sectioned for hematoxylin and eosin (H & E) staining.

## SUPPLEMENTARY FIGURES AND TABLES


