# Supplementary material for: 5-hydroxytryptamine receptor (5-HT1DR) promotes colorectal cancer metastasis by regulating Axin1/β-catenin/MMP-7 signaling pathway
Source: Oncotarget. 2015 Jul 17;6(28):25975–87. doi: 10.18632/oncotarget.4543 (PMC4694879; doi:10.18632/oncotarget.4543)
Supplement: Supplementary file 1 [file oncotarget-06-25975-s001.pdf]

SUPPLEMENTARY FIGURES AND TABLES

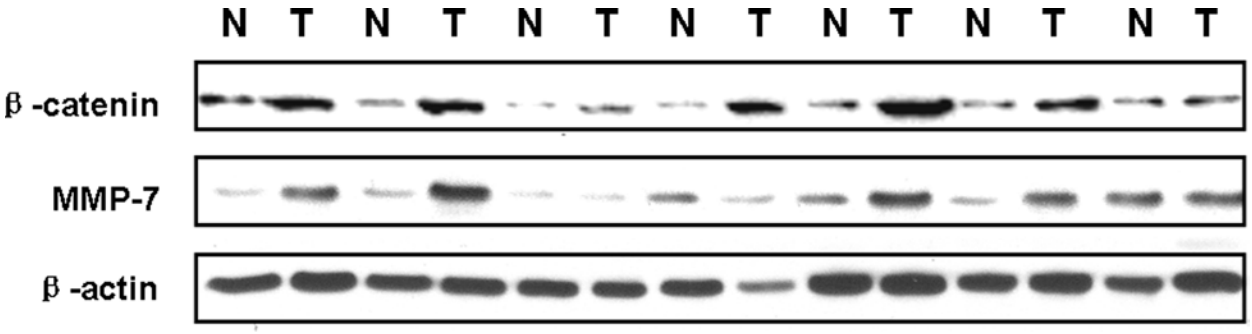

Supplementary Figure S1: β-catenin and MMP-7 expression in CRC tissues. Western blot analysis performed to detect the expression of β-catenin and MMP-7 in 7 pairs patient CRC tissues-matched normal tissues. The samples are higer-expressed of 5-HT_{1D}R.

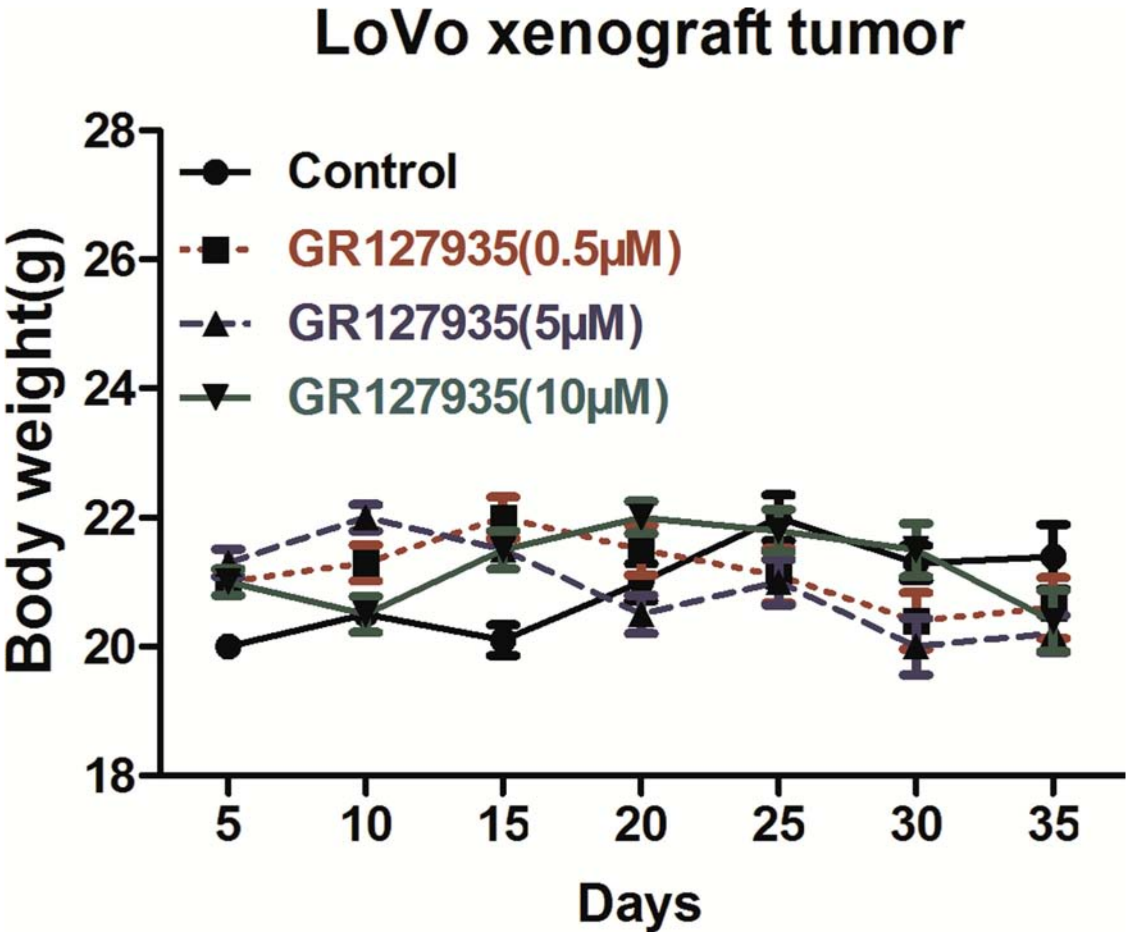

Supplementary Figure S2: Body weights of mice. Body weights of mice xenografted with LoVo cells and treated with 5-HT_{1D}R antagonist (GR127935) in a does-dependent manner.

Supplementary Table S1. Patient information for colorectal metastasis cancer

Characteristics	$5\text{-}HT_{1D}R$ expression		$5\text{-}HT_{3C}R$ expression		$5\text{-}HT_4R$ expression	
	Negative (*n* = 22)	Positive (*n* = 68)	Negative (*n* = 37)	Positive (*n* = 53)	Negative (*n* = 43)	Positive (*n* = 47)
Gender						
Male	18	42	30	30	27	33
Female	**4**	**26**	**7**	23	16	14
Age/year						
>60	10	39	19	30	33	16
≤60	12	29	18	23	10	31
Tumor size d/cm						
<3	3	2	3	2	3	2
≥3 ~ <5	11	33	19	25	22	22
≥5	8	33	15	26	18	23
TNM staging						
I	7	6	12	1	9	4
II	13	46	21	38	31	28
III	2	16	4	14	3	15
IV	0	0	0	0	0	0
AFP (ng/mL)						
<200	5	15	10	10	9	11
≥200	17	53	27	43	34	36
CEA (ng/mL)						
<20	4	9	6	7	6	7
≥20	18	59	31	46	37	40
Nodal status						
Positive	17	59	30	46	37	39
Negative	5	9	7	7	6	8

Supplementary Table S2. PCR primers

Gene	Forward primer	Reverse primer
Wnt2	TGAGTCTCACCACTAGCCGCA	ACTGGGAATCAGCCAGGGAGGGT
MMP-7	TGTATGGGGAACTGCTGACA	GCGTTCATCCTCATCGAAGT
Akt	ATGAGCGACGTGGCTATTGTGAAG	GAGGCCGTCAGCCACAGTCTGGATG
MAPK10	CAGCTCTCTAAATTGACTCAG	CCAATGGTTGGTTCACTGCAG
APC	ATTGTTATTAATTTTTTTGTTTGTTGGG	CGAACTACACCAATACAACCACATATC
VEGF	CTCTACCTTCCACCATGCCAAG	AGACATCCATGAACTTCACCACTTC
p53	GTCTACCTCCCGCCATAA	CATCTCCCAAACATCCCT
SOX9	ATCTGAAGAAGGAGAGCGAG	CAAGCTCTGGAGACTGCTGA
β-catenin	AGGCATCCTCACCCTGAAGTA	CACACGCAGCTCATTGTAGA
COX-2	ATGCTGACTATGGCTACAAA	TGATGCGTGAAGTGCTG
CXCR4	GCCAACGTCAGTGAGGCAGATG	GAGGATGACTGTGGTCTTGAGG
TICAM-1	GGGAATTCATAATGGGTATCGGGAAGTC	GGCTGCAGGTTATATGTTTCATCTCAGGC
E-cadherin	GAACGCATTGCCACATA	GCACCTTCCATGACAGAC
ABCG2	CCCTGAGAAACTCCTTGAA	TGCTGGATAGTCGGTGC
ABCB1	TAATGCGACAGGAGATAGG	TGCCATTGACTGAAAGAAC
Bcl-xl	GGTGCCACCTGTGGTCCACCTG	GTTCACTGTTGGCCCAGATAGG
GAPDH	GGTCGTATTGGGCGCCTGGTC	TGACGGTGCCATGGAATTTGCCA
